# Adherence to Acromegaly Treatment and Analysis of the Related Factors—A Real-World Study in Bulgaria

**DOI:** 10.3390/pharmaceutics15020438

**Published:** 2023-01-28

**Authors:** Maria Kamusheva, Silvia Vandeva, Konstantin Mitov, Alexina Parvanova, Marina Pesheva, Nikolay Ganov, Yanitsa Rusenova, Lyubomir Marinov, Violeta Getova, Atanaska Elenkova, Guenka Petrova

**Affiliations:** 1 Department of Organization and Economics of Pharmacy, Faculty of Pharmacy, Medical University of Sofia, 1000 Sofia, Bulgaria; 2 Department of Endocrinology, USHATE “Acad. Ivan Penchev”, Medical University of Sofia, 1000 Sofia, Bulgaria; 3 Department of Pharmacology, Pharmacotherapy and Toxicology, Faculty of Pharmacy, Medical University of Sofia, 1000 Sofia, Bulgaria

**Keywords:** adherence, acromegaly, Bulgaria

## Abstract

The purpose was to assess the level of medication adherence (MA) and related factors among individuals with acromegaly. The secondary goal was to assess the quality of life of patients and whether and how it correlates with the level of adherence. A prospective one-year study was conducted among patients with acromegaly diagnosed, treated, and monitored in the reference center for rare endocrine diseases in Bulgaria in 2021. Clinical data, patients reported outcomes, and health economics data were collected to define the predictors of non-adherence to medicines. Medication adherence level was assessed through a free Morisky–Green 4-item questionnaire. A total of 179 patients with acromegaly were observed. Approximately 62% were female, 50% were between 41 and 60 years, and the mean age at diagnosis was 40.4 years. The response rate to the questionnaires was 53% (*n* = 95; mean age 53.5 years, 73% female and 26% male). Patients with high levels of MA reported higher median values for the 36-Item Short Form Health Survey (SF-36) in comparison with those with low levels: 65.5 vs. 48.5 (*p* = 0.017). Similar results for EQ-5D-3L (3-level EuroQol 5D version) values and the level of MA were found: 0.656 vs. 0.796 (*p* = 0.0123). A low level of adherence was revealed in 34.7% of the patients, with no difference among different age groups. A significant positive determinant for adherence was years lived with acromegaly (OR = 5.625, 95% CI 1.7401–18.1832, *p* = 0.0039), as shorter duration was related to higher odds for high level of adherence. The current study demonstrates the importance of MA assessment for patients with acromegaly in Bulgaria. The medication adherence to the prescribed therapy among the observed group of patients with acromegaly varied as the percentage of adherent patients was around 65%. Still, there are low-adherent patients, and the responsible factors should be further investigated.

## 1. Introduction

Pharmacotherapy results of rare diseases treatment depend on numerous factors from both patients’ and healthcare specialists’ sides [1]. Even though the treatment of acute and chronic conditions is often based on fundamentally different approaches, the extent to which patients comply with prescribed guidance is proven a determining factor for the success of therapy [2,3]. Medication adherence, as the level at which patients actively follow the given recommendations from the prescriber, is defined as one of the crucial elements of rational medicines utilization [4,5]. Moreover, some studies suggest that the higher the adherence, the lower the risk of drug-related problems [6,7,8]. Therefore, adherence to therapy is a key prerequisite for positive treatment outcomes and lower safety risk, which is especially important for patients that require long-term health care.

Achievement of a satisfying level of adherence is a challenge for healthcare professionals, with some researchers pointing it out as one of the most significant problems in addressing the self-management of chronic diseases [9]. Many factors have an influence on the level of adherence, some of which are related to concomitant diseases (such as mental and nervous system comorbidities). Patient involvement in the decision-making process during therapy, advanced age, and level of health literacy are just a few points to be considered in understanding the problem [2,10].

Acromegaly is a rare endocrine disease for which early diagnosis and optimal adherence to therapy are identified as key factors for the achievement of long-term control [11]. Newer therapy options include long-acting depot medications, which require a reduced number of injections and are associated with better adherence due to the reduced burden on patients [9,10]. Literature data about adherence to the new therapeutic options for rare diseases therapy are scarce.

Identifying the causes leading to insufficient adherence is an important step in the management of therapy outcomes, as improvement of adherence can lead not only to better therapy results but to an overall reduction in healthcare costs as well [7,12,13,14]. This is of special importance in therapeutic areas where such studies are limited, one of them being the long-term management of acromegaly [15]. Considering that the National Health Insurance Fund in Bulgaria covers the acromegaly treatment fully, the level of co-payment is very low due to the mechanism of internal reference pricing, and the financial reason could not be a limitation for an adequate level of treatment, our interest was to identify what the level of adherence to acromegaly treatment is and which the relevant influencing factors are.

The purpose of this study is to assess the level of medication adherence (MA) and related factors among individuals with acromegaly. The secondary goal was to assess the quality of life of patients and whether and how it correlates with the level of adherence.

## 2. Materials and Methods

### 2.1. Study Design

A prospective one-year study was conducted among patients with acromegaly diagnosed, treated, and monitored in the reference center for rare endocrine diseases in Bulgaria in 2021. The reference center admits patients from the whole country area and is following almost all Bulgarian patients with acromegaly.

### 2.2. Setting, Patient Recruitment, and Sample Size

All patients treated for acromegaly who were on pharmacotherapy and gave their informed consent to participate were included. According to the legislation requirement, before admittance to the clinic, each patient should fill in a declaration and informed consent to treatment. Up to now, the hospital is the only pituitary center in the country prescribing somatostatin analogs and pegvisomant for the treatment of acromegaly, which allowed us to invite for participation all Bulgarian patients on these medications. The study period was determined between 1 January 2021 and 31 December 2021.

### 2.3. Characteristics and Cost of Therapy

Clinical data were gathered from the patients’ medical records (data in the electronic database and patients’ paper files). The collected data included demographic characteristics (age, gender, place of birth); clinical data (acromegaly duration; year of diagnosis, insulin-like growth factor 1 (IGF-1) and growth hormone (GH)); pharmacotherapy and other therapy: surgery and/or radiotherapy; comorbidity; healthcare resources utilization (hospitalizations per year; medicines utilized); days off work due to acromegaly extracted from the patients’ records.

Healthcare costs such as direct (for acromegaly therapy, hospitalization, concomitant medications per year) and indirect (days out of work for the working age population) costs were calculated for a one-year period (2021) using a micro-costing approach. Reimbursement levels and the price per unit paid by the NHIF for all included in the Positive Drug List medicines for patients with acromegaly were considered. The unit medicines costs were obtained from the Positive Drug List. On the basis of the number of hospital days for every patient and gross domestic product per capita for 2021, we calculated the indirect costs (days out of work (absenteeism)) by applying the human capital approach formula. These variables (demographic, clinical, quality of life, and economic outcomes) were used to define the predictors of non-adherence to medicines among the included patients with acromegaly in the study.

### 2.4. Assessment of Medication Adherence and Quality of Life

For the purposes of the current study, we used the Seventh Framework Program for research and technological development (FP7) funded ABC project (Ascertaining Barriers to Compliance: policies for safe, effective, and cost-effective use of medicines in Europe) commonly applied definition from 2012. ‘Adherence to medications’ is the process by which patients take their medication as prescribed, further divided into three quantifiable phases: ‘Initiation’, ‘Implementation’, and ‘Discontinuation’ [16]. We applied the free Morisky–Green 4-item questionnaire to define the level of adherence to therapy for the main disease among patients with acromegaly. The questionnaire consists of four questions with two options to answer (“yes” or “no”) [17]. The scale is from 0 to 4, as three levels of medication adherence based on this score were suggested: high (0 points), medium (1–2 points), and low (3–4 points) adherence [18]. It was distributed among the patients with acromegaly willing to participate in the survey. According to the available literature, only an 8-item Morisky Medication Adherence Scale (MMAS-8) questionnaire was applied to Bulgarian patients with cardiovascular diseases and chronic myeloid leukemia [19,20]. However, we chose to apply Morisky–Green 4-item questionnaire. This questionnaire is due to its availability as a free instrument and considering its length, consistency, and understandability.

The adherence was evaluated towards the pharmacotherapy recommended by the national pharmacotherapeutic guidelines that include monotherapy with dopamine agonists (DA), SSA, Growth hormone receptor antagonist (GHRA), and combination therapy SSA + GHRA, GHRA + DA, SSA + GHRA + DA, and SSA + GHRA [21]. Diagnostics and treatment of patients with acromegaly were in accordance with the national guideline on endocrinology and metabolic diseases from 2019, based on the international and the European endocrine society guideline [22,23]. Management of these patients was also consistent with the updated recommendations of the Pituitary society from 2021 [24]. In addition to the level of adherence to prescribed therapy, quality of life (QoL) was measured by two different instruments (EQ-5D-3L (3-level EuroQol 5D version) and the 36-Item Short Form Health Survey (SF-36)). Their structure is explained elsewhere [25,26], but in general, the EQ-5D-3L is a unidirectional measure of the QoL providing the utility between 0 (death) and 1 (perfect health), and SF-36 is a multidimensional QoL measure providing the utility between 0 (death) and 100 (perfect health) for 7 dimensions of the QoL. All questionnaires (SF-36, EQ-5D and Morisky-Green 4-item) in Bulgarian and in English language are available as Supplementary file.

The patients were questioned with the three instruments ones during the whole period of observation when they were invited for monitoring in the hospital.

### 2.5. Statistics

A set of statistical methods were applied to describe and assess the correlations among data of interest: descriptive statistics, non-parametric analysis—Kruskal–Wallis and Mann–Whitney test, odds ratio test, and regression analysis. MedCalc statistical software version 16.4.1 for biomedical research was used. Descriptive statistics were applied to present the basic characteristics of the patients: gender, age, birthplace, age of diagnosis, type of therapy, quality of life, level of adherence, etc. Comparisons between the median values of different patients’ groups’ characteristics were performed by the Kruskal–Wallis test. Non-parametric Mann–Whitney test for independent samples was applied to compare the median values for age at diagnosis, IGF-1 levels, GH-levels for men and women, etc.

Clinical, demographic characteristics, quality of life, and cost variables were used to define the predictors of non-adherence to medicines among the included patients with acromegaly in the study. The odds ratio (OR) was used to assess the association between these variables. It was estimated using logistic regression adjusted for confounding factors (*p* < 0.05 expressed statistical significance). The odds ratio (OR) of being with high or low adherence is calculated with logistic regression for every demographic characteristic of the observed patients (gender, age, etc.).

### 2.6. Ethics

All patients with acromegaly admitted for a regular examination that agreed to participate were recruited for this study. All patients, with no exception, provided signed written informed consent at their admission authorizing the use of their anonymized (pseudonymized) data for scientific purposes. The ethical committee of the hospital approved the study.

## 3. Results

### 3.1. Demographics

All patients admitted for regular examination (*n* = 179) in the University Hospital “Acad. Ivan Penchev”, Sofia, Bulgaria, in 2021 were invited to participate and fill out the questionnaires. Significantly more women were recruited, as they represented 62.0% of the sample (*p* = 0.0013). Only 16.2% of the patients live in rural areas, which is almost 4 times less than those from urban areas (*p* < 0.0001). The distribution of age groups was not normal (*p* = 0.0076 < 0.05) but close to symmetrical. The mean age was 54.64 years (95% CI 52.714–56.571; SD = 13.07) (Table 1).

### 3.2. Clinical Characteristics

The mean age of diagnosis was 40.4 years (95% CI 37.345–43.486; SD = 12.39) for males and 42.7 years (95% CI 40.468–45.07; SD = 11.8307) for females. No statistically significant difference between the median age at diagnosis for acromegaly for both genders (*p* = 0.1382) and place of birth (*p* = 0.1580) was revealed. The median levels of IGF-1 for males and females during the previous hospitalization were 23.10 nmol/L (95% CI 18.615–46.341) and 20.80 nmol/L (95% CI 17.163–24.225), respectively (*p* = 0.1208). During the current examination, the IGF-1 levels were higher (27.1 (95% CI 21.375–30.749) and 23.65 (95% CI 20.673–26.913), respectively), but no statistical significance was found.

The levels of IGF-1 during the previous and during the current examination were compared as the patients were separated into several subgroups based on the type of therapy (monotherapy or combination therapy). The median values for the subgroups were different but not statistically significant, probably due to the low number of patients in the subgroups (Table 2 and Table 3).

**Figure 1:**
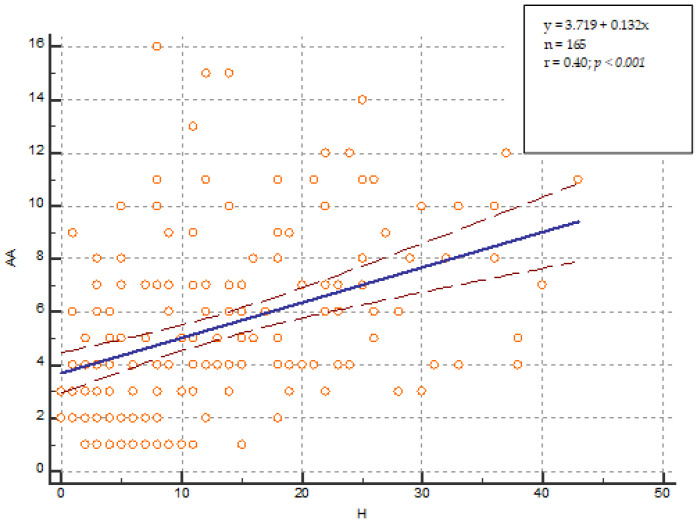
Linear correlation analysis. H—years lived with acromegaly. AA—number of concomitant diseases.

Women were diagnosed with more concomitant diseases than men: 5 (95% CI 4–7) vs. 4 (95% CI 3–5) (*p* = 0.0241). The longer duration of acromegaly was related to more concomitant diseases. The coefficient of determination R^2^ is equal to 0.1615, which means that 16% of variations in years lived with acromegaly are in correlation with the number of concomitant diseases. The accuracy of the model is set with a Residual standard deviation = 3.0521. Both coefficients of the linear regression (Intercept and Slope) are statistically significantly different from zero because their *p*-values are less than 0.05. The model adequately describes the relationship between both variables because the result of the F-test is statistically significant—*p* < 0.0001. It can be used to predict the average values of one variable for a given value of the other variable (Figure 1).

### 3.3. Pharmacotherapy and Costs

Transsphenoidal adenectomy (TSA) was applied among 87.2% of the patients (*n* = 156). Forty-three patients (*n* = 43; 24%) underwent transsphenoidal resurgery. Transcranial adenomectomy (TCA) after TSA was applied in 11 patients (6% of all cases) with invasive pituitary tumors with large extrasellar portions inaccessible for transsphenoidal approach. Radiotherapy was performed in 30 patients (17.1%), in 28 of them (15.6%)—after TSA. In all patients included in this study, medical specialists (mostly nurses) applied SSA. Telemedicine was extremely rare for these patients, and it was provided only during the short COVID-19 pandemic lockdown periods. Our patients could make contact by telephone and discuss their issues with endocrinologists and nurses engaged with their management in the tertiary center.

The monthly costs paid by the National health insurance fund (NHIF) for patients on monotherapy (SSA or DA or Pegvisomant) (*n* = 99) were lower than for those on combination therapy (*n* = 36): 659.45 BGN (95% CI 659.45–989.29) vs. 6472.68 BGN (95% CI 5057.797–9069.88) (*p* < 0.0001).

**Figure 2:**
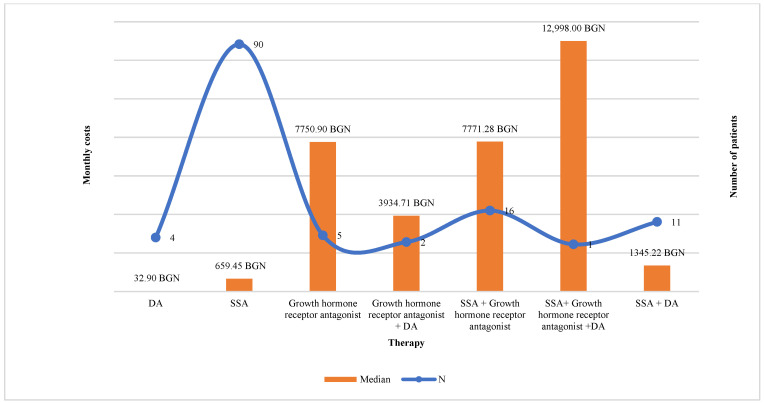
Monthly costs by NHIF for acromegaly therapy.

**Figure 3:**
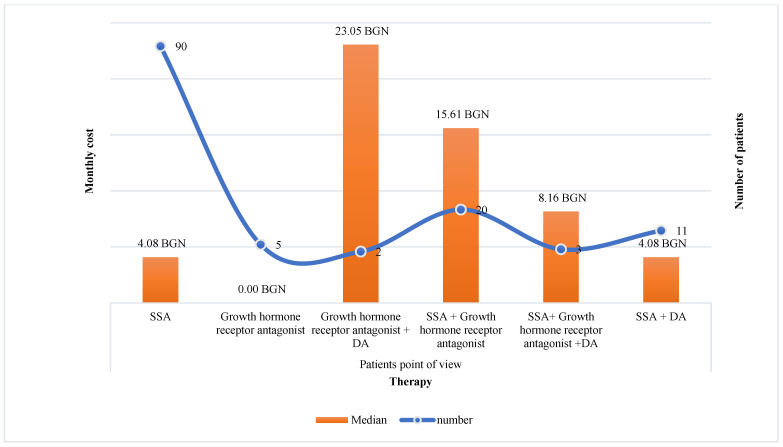
Co-payment for acromegaly therapy per month per patient.

The highest was the number of patients on treatment with SSA (*n* = 90), followed by combination therapy SSA + GHRA (*n* = 16). The median monthly costs paid by NHIF were the highest for patients treated with triple combination (SSA + GHRA + DA) 12,998.00 BGN, dual combination SSA + GHRA 7771.28 BGN (95% CI 6010.599–9069.88), and monotherapy with GHRA 7750.98 BGN (*p* < 0.000001) and the lowest were for dopamine agonists 32.90 BGN (Figure 2). Out-of-pocket payment was logically lower for patients on monotherapy in comparison with combination therapy 4.08 BGN (95% CI 4.08–4.08) vs. 8.16 BGN (95% CI 6.768–23.05). Therapies with SSA + GHRA and with GHRA + DA were related to the highest financial burden from the patients’ point of view, 15.61 BGN (95% CI 8.16–31.21) and 23.05 BGN, respectively, per month (*p* < 0.05) (Figure 3).

The median monthly costs paid by NHIF for all 3 groups of patients with low, medium, and high levels of adherence were not significantly different: 824.37 BGN (95% CI 659.45–1318.90), 850.69 BGN (95% CI 659.45–1318.90), and 989.18 BGN (95% CI 659.45–5145.75) (*p* = 0.8381). Co-payment was also similar among these three subgroups: 4.08 BGN (95% CI 4.08–8.16). Indirect costs among all patients with different levels of adherence were 464.29 BGN (95% CI 386.91–541.67). The monthly costs for concomitant diseases paid by NHIF were 7.64 BGN, 14.88 BGN, and 6.36 BGN for the observed subgroups based on their level of adherence. The co-payment for the treatment of concomitant diseases was 25.45 BGN, 45.73 BGN, and 20.07 BGN, respectively.

### 3.4. Medication Adherence Level and Determinants of Medication Adherence

The response rate to the MA questionnaire was 53% (*n* = 95). The mean age was 53.5 years, as 74% were female and 26% were male) with mean age at diagnosis 41.5 years. The patients’ characteristics of the respondents are presented in Table 4. All operated patients underwent TSA. Radiotherapy was applied in 16 out of 95 patients (4 males and 12 females). The median levels of IGF-1 for males and females during the previous hospitalization were 28.23 nmol/L [SD = 20.81] and 30.77 nmol/L [SD = 25.39], respectively (*p* = 0.65). During the current examination, the IGF-1 levels were higher for men, 42.21 [SD = 42.19], and lower for women, 28.13 (95% CI 20.673–26.913), but no statistical significance was found. Due to the COVID-19 pandemic measures, some of the visits of our patients with acromegaly were shorter than usual, which could partly explain the lower response rates. Poor level of adherence was indicated for 34.7% (*n* = 33), medium for 40% (*n* = 38), and high for 25.3% (*n* = 24). The mean age of patients with poor adherence was 57.03 years (95% CI 52.831–61.23), 60.18 years (95% CI 55.472–64.897) for those with medium level and 52.5 years (95% CI 46.99–58.01) for high-level adherent patients. However, no statistically significant difference in the mean age in the groups with different levels of adherence was detected (*p* = 0.088). Monotherapy was prescribed to most of the patients (64.2%, *n* = 61). The odds of being adherent in the case of monotherapy were equal to the group on combination therapy (OR = 0.6261; 95% CI 0.2936–2.766, *p* = 0.8557) (Table 5). Due to the low number of patients on different therapies, a comparison between the subgroups to demonstrate which therapy is related to the highest level of adherence could not be presented. Only the sample size of the patients treated with SA and SA + pegvisomant was large enough to analyze the odds of being adherent. Among patients treated with SSA, 61.4% were with medium or high level of adherence. From those on combination therapy (SSA + pegvisomant) (*n* = 8), only 25% reported a low level of adherence. A lack of correlation between TSA and level of adherence was revealed (*p* = 0.3346). Due to the lower response rate to the questionnaires, the analysis for resurgery and other types of surgery was not possible to be performed.

A low level of adherence was reported from more men than women (38% vs. 33%, *p* = 0.66491) as well as from those living in rural than in urban areas (50% vs. 31%, *p* = 0.1291) but without statistical significance. Therefore, it should be stated that there is no statistical reason to claim that the level of adherence differs between different genders and regions. A significant positive determinant for adherence was years lived with acromegaly (OR = 5.8594, 95% CI 11.8176–18.8891, *p* = 0.0031)—shorter duration is related to higher odds for the high level of adherence (Table 5). Kruskal–Wallis test confirmed that there is a correlation between years lived with acromegaly and the level of adherence: patients who lived more with acromegaly reported a low level of adherence (median 15.5 years (95% CI 11.998–22.002)) whereas those diagnosed 5 years ago (95% CI 4–13.302) were with high level of adherence (*p* = 0.0268).

### 3.5. Quality of Life

**Figure 4:**
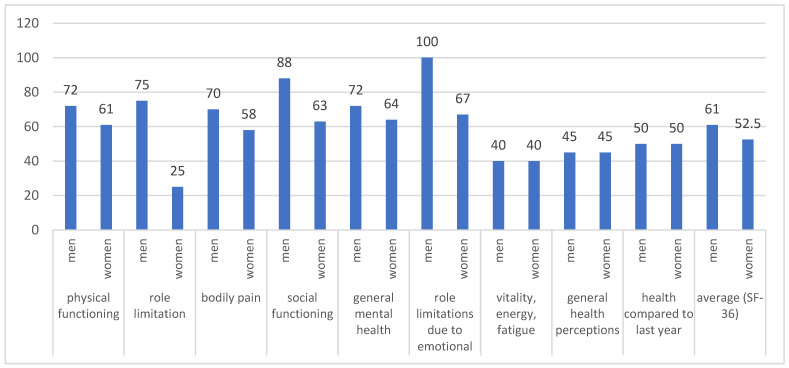
SF-36 scales for both genders.

**Figure 5:**
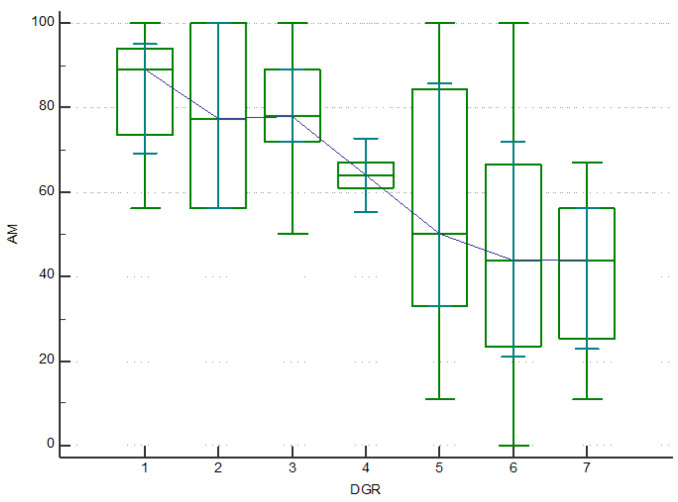
Physical functioning among different age groups. DGR—age groups. AM—physical functioning.

The completion rate of both quality-of-life questionnaires was the same as the rate of the MA questionnaire. The median values for most of the scales of the SF-36 questionnaire were higher for men than women (*p* < 0.05) (Figure 4). Logically, the higher age was related to lower median values for physical functioning (Figure 5) (linear regression model with a coefficient of determination R^2^ = 0.2467, y = 101.365 + (−0.794) x, *p* < 0.001). In contrast, the median values for EQ-5D were similar for both men and women, 0.725 (95% CI 0.691–0.848) vs. 0.691 (95% CI 0.648–0.758), respectively.

Patients with high levels of medication adherence reported higher median values for SF-36 in comparison with those with low levels of medication adherence: 65.5 vs. 48.5 (*p* = 0.017). Similar results for EQ-5D values and the level of medication adherence were found: 0.656 vs. 0.796 (*p* = 0.0123) (low vs. high level). No difference in QoL between patients with and without surgery (0.725 vs. 0.691, *p* = 0.6414) was found.

## 4. Discussion

To the best of our knowledge, this is the first study focused on medication adherence and predicting factors for non-adherence among patients with acromegaly in Bulgaria performed in real-world clinical practice through an MA assessment questionnaire. A previously conducted national-based study by Kamusheva et al., 2021 [27] analyzes the need for and importance of a treatment adherence guideline for patients with acromegaly, the possibilities for its development and implementation in Bulgaria and presents the results from a pilot study on MA among hospitalized Bulgarian patients with acromegaly. The authors assessed patients’ adherence based on patients’ records of the regular consumption of prescribed therapy and the reasons for non-adherence without using any questionnaires.

There is no validated and specific questionnaire or other methods for measuring medication adherence in the acromegaly population, neither worldwide nor in Bulgaria. In the current study, we applied the MA test as only MMAS-8 was validated for Bulgarian patients with other chronic and lifelong diseases such as cardiovascular, pulmonary disorders, and chronic myeloid leukemia [19,20].

Demographic characteristics identified in the current study confirmed previously published national-based results [3]. More women than men were hospitalized 62% vs. 38% in comparison with the study from 2018—67% vs. 33%. In both studies prevailed patients between 40 and 60 years of age (50% in 2021 vs. 45.5% in 2018), and the mean age at diagnosis is similar—40.4 years vs. 40.7 years, respectively. There was no statistically significant difference in IGF-1 levels in comparison with the previous monitoring examination revealed in both years of observation (2018 and 2021). The study confirmed that the longer duration of the diseases is related to higher number of concomitant diseases. Moreover, we developed a model that describes the relationship between the duration of acromegaly and the number of concomitant diseases in patients with acromegaly that could be used for further predictions and analysis. Our results were in accordance with other published studies stating that patients with acromegaly had more comorbidities than those without acromegaly, which proves the effect of acromegaly on the general health condition [28]. According to other large studies, concomitant diseases among patients with acromegaly are factors leading to an increase in mortality rates [29]. Thus, consecutive detailed follow-up analyses and control improvement of both main and concomitant diseases in this group of patients could be defined as the main approaches how to influence the mortality and morbidity levels.

The general questionnaires, such as EQ-5D and SF-36, have confirmed that quality of life is significantly impaired in patients with acromegaly, as the values were far below the maximum possible values. We revealed a lack of difference in utility values between both genders measured by EQ-5D—around 0.7 as the higher age was related to lower values for physical functioning among all patients. As other authors stated, QoL in patients with acromegaly could be determined by various factors depending on the level of disease control [30,31,32]. However, we focused not on the key factors influencing QoL, such as age, active disease, and type of therapy, as it could be the focus of further, more comprehensive study, but on the medication adherence level and how it influences the patients’ well-being and utility. Our study confirmed that patients with high MA levels reported higher utility values for both questionnaires applied: (1) median values for SF-36 were 65.5 for patients with high levels of MA and 48.5 for those with low levels of MA; (2) median values for EQ-5D—0.796 and 0.656, respectively for both levels of MA. Our study is in accordance with other findings for other groups of patients with diabetes and hypertension that adherent patients had significantly higher QoL and health scores [33,34]. Therefore, by aiming at the improvement of MA in patients with acromegaly, we could improve patients’ quality of life, treatment satisfaction, and trust in the healthcare system [35,36,37].

In accordance with the study conducted in 2018 [3], patients treated with SSA prevailed as they represented 69% of all observed patients, followed by combination therapy SSA plus GHRA (15%). The financial burden was lower for the patients in comparison with the payer’s point of view for all types of treatment. Investigating the relationship between the level of MA and the monthly costs, we have not revealed any significant differences in the costs among the different MA-level groups. The level of co-payments and higher medication costs negatively influence the MA [38]. However, considering the high level of reimbursement of acromegaly therapy in the country, we might conclude that the financial aspects are not an influencing factor on MA among patients with acromegaly.

Since acromegaly is a chronic disease requiring long-term treatment and the expected clinical improvement is closely related to the type of therapy and the persistence to treatment recommendations, it is crucial to follow up and improve the level of adherence. In their study, Cámara et al. stated that despite the level of adherence to pegvisomant treatment in Spain being high, it should be closely monitored in order to improve the treatment effectiveness in real-world clinical practice [39]. According to the data shown in the results section, we have not included patients with no current treatment, but they are monitored on a regular basis. We did not evaluate their compliance with the visits due to the focus only on medicines. It could be a goal of further study to evaluate and comply with the overall treatment process. Our study found that only 34.7% of the patients were with low levels of adherence, with no difference among different age groups. Due to the low number of patients in different therapeutic groups, it was not possible to define which therapy is associated with better MA. The relative share of patients on SSA and on combination therapy (SSA + pegvisomant) with low levels of MA was 38.6% and 25%, respectively. No other similar studies applying Morisky–Green test for patients with acromegaly MA assessment were found. Most of the authors used the medication possession ratio as the main method for measurement. A study conducted in 2017 found that treatment adherence, assessed using medication possession ratio, was similar for injectable SSA being 87% or greater. Lanreotide was associated with greater persistence than octreotide and a lower risk of discontinuation [40]. A systematic review performed in 2020 reported eleven studies examining the level of adherence, compliance, and persistence among patients with acromegaly [3]. The review confirmed that the main methods for assessing adherence were medication possession ratio (MPR) (≥80%), number of injections, treatment duration, questionnaires, prescription-refill records, counting of missed doses, and the adherence rates vary between 60.7–92.1% for pegvisomant, 87% for lanreotide depot, and 89% for octreotide LAR [3]. We should emphasize that the only issue regarding the discontinuation of treatment which could be met by Bulgarian patients with acromegaly is the lack of medication for their diseases due to drug shortages. However, during the current study period, the patients did not report such an issue. The results of our study about the relation between the duration of the diseases and level of adherence were in accordance with those revealed in the systematic review—shorter duration of acromegaly was related to higher odds for high level of adherence which could be explained with more severe symptoms in the first years of diagnosis. A lot of effort should be put into assessing and regularly reassessing the level of medication adherence during routine clinical practice so as to achieve the therapeutic outcomes, and overall well-being of patients, improve their quality of life, and reduce the risk of complications.

Several strengths of the study might be highlighted. First, this is the first study focusing on the MA and influencing factors among patients with acromegaly in Bulgaria. Second, it encompasses patients with acromegaly who were diagnosed, treated, and followed up at the reference center for rare endocrine diseases in the country. Third, the patients were included from the only pituitary center in the country supporting a computerized register for patients with acromegaly, which allowed us to include all patients in the country.

Using a non-validated questionnaire for MA assessment could be highlighted as one of the main limitations of the current study. However, it could be used as an initial validating study for the purposes of further similar analyses. Another limitation comes from the lack of strict analysis of the factors that influence MA. MA test aims to identify whether the patients adhere to the therapy or not without focusing on the determinants of MA. Further study using a specifically formulated questionnaire about the predictors of MA should be conducted. The low response rate to the questionnaires, which could be explained by the COVID-19 pandemic situation, did not allow us to collect more data and analyze how resurgery and other types of surgery, except TSA, are influencing MA and QoL. Moreover, we have not collected detailed information for tumor remnants characteristics such as stable or progressive, cavernous invasion, presence of optic chiasma syndrome, pituitary failure substitutive treatment, or colon cancer, and for the type of concomitant diseases. We have not evaluated the probable adverse drug reactions and their influence on adherence to medication therapy. Considering the applied statistics and the analysis based on concurrent data collected over the same period, we should highlight as a limitation that the association between cost variables and adherence should be interpreted with caution. Another strong limitation is the collection of data by questionnaires which might influence the analysis and conclusions due to recall bias as well as the lack of any quality control measures during data collection. Moreover, we have not investigated and analyzed another important factor—parallel trade with medicines. As one of the greatest issues in the country is the parallel trade of medicinal products that leads to drug shortages even for patients with acromegaly, further studies analyzing this effect and related challenges in how to cope are needed.

## 5. Conclusions

The current study demonstrates the importance of medication adherence assessment for patients with acromegaly in Bulgaria. The medication adherence to the prescribed therapy among the observed group of patients with acromegaly varied as the percentage of adherent patients was around 65%. The study shows that the higher quality of life and the shorter duration of the disease, the higher the MA levels are. Still, there are low-adherent patients, as the related factors should be further investigated. Moreover, as it is the first study in Bulgaria applying a questionnaire to evaluate MA among patients with acromegaly, consecutive follow-up comparative studies to confirm the correlation between years lived with acromegaly, quality of life, and the level of adherence are highly recommended.

## Figures and Tables

**Table 1:** Demographic characteristics of patients with acromegaly (*n* = 179).

Category	Subgroup	Number [Distribution in %]
Gender	Male	68 [38%]
	Female	111 [62%]
Region	Village	29 [16.2%]
	Town/city	150 [83.8%]

**Table 2:** IGF-1 levels and type of therapy by number of included medicines in the therapeutic scheme.

Type of Therapy/IGF-1 Levels in nmol/L	No Treatment (*n*_1_ = 12; *n*_2_ = 39)	Monotherapy (*n*_1_ = 48; *n*_2_ = 96)	Combination Therapy (*n*_1_ = 22; *n*_2_ = 34)	*p* Value
IGF-1 levels at the previous hospitalization	27 (95% CI 18.142–32.044)	18.8 (95% CI 15.244–24.035)	21.5 (95% CI 18.53–38.66)	0.4567 *
IGF-1 levels at the current hospitalization	23.6 (95% CI 18.96–34.508)	23.8 (95% CI 20.492–28.155)	26.65 (95% CI 19.856–28.939)	0.9591 *

* not statistically significant. *n*_1_—number of patients at the previous hospitalization; *n*_2_—number of patients at the current hospitalization in 2021.

**Table 3:** IGF-1 levels and type of therapy by mechanism of action.

Type of Therapy/IGF-1 Levels in nmol/L	DA (*n*_1_ = 3; *n*_2_ = 4)	SSA (*n*_1_ = 42; *n*_2_ = 87)	GHRA (*n*_1_ = 3; *n*_2_ = 5)	GHRA + DA (*n*_1_ = 0; *n*_2_ = 2)	SSA + GHRA (*n*_1_ = 14; *n*_2_ = 19)	SSA + GHRA + DA (*n*_1_ = 1; *n*_2_ = 3)	SSA + DA (*n*_1_ = 7; *n*_2_ = 10)	No Treatment (*n*_1_ = 12; *n*_2_ = 39	*p* Value
IGF-1 levels at the previous hospitalization	15.50	18.65 (95% CI 14.092–23.508)	46.40	-	18.80 (95% CI 16.135–27.999)	70.20	28.90 (95% CI 10.499–69.222)	27.00 (95% CI 18.142–32.044)	0.264 *
IGF-1 levels at the current hospitalization	19.65	23.10 (95% CI 19.788–27.216)	47.10	22.65	26.60 (95% CI 14.735–29.389)	13.80	30.35 (95% CI 20.48–70.918)	23.60 (95% CI 18.96–34.508)	0.2124 *

* (not statistically significant). *n*_1_—number of patients at the previous hospitalization; *n*_2_—number of patients at the current hospitalization in 2021. The IGF-1 levels were available for some of the observed patients.

**Table 4:** Characteristics of the respondents.

	Number [%]	Years [SD]	Type of Surgery	Number of Patients with TSA [%]	Number of Patients with Radiotherapy [%]	Urban Region	Years Lived with Disease [SD]	Average Number of Concomitant Diseases	Type of Therapy	Utility Values [SD]
Male	26 [27.4%]	53.54 [14.41]	TSA *	25 [96.2%]	4 [15.4%]	77%	13.76 [10.66]	5	18—SSA 3—SSA + DA 5—No treatment	0.74 [0.24]
Female	69 [72.6%]	58.5 [12.84]	TSA	69 [100%]	12 [17.4%]	82.6%	14.4 [11.14]	7	40- SSA 8–SSA + pegvisomant 5-SSA + Da 12-no treatment 2-pegvisomant 2-DA	0.70 [0.22]

* TSA—Transsphenoidal adenectomy.

**Table 5:** Logistic regression results.

Determinant	High Level of Adherence (*n*)	Medium Level of Adherence	Low Level of Adherence (*n*)	OR, 95% CI, *p* (for High and Low Levels) **
Gender Male Female	7 17	9 29	10 23	OR = 0.9471, 95% CI 0.2994–2.9955 *p* = 0.9262
Birth place Village City/Town	5 19	4 34	9 24	OR = 0.7018 95% CI 0.2015–2.44 *p* = 0.57
Years lived with acromegaly ≤10 years >10 years	15 8	18 19	8 25	OR = 5.8594 95% CI 1.8176–18.8891 *p* = 0.0031 *
Type of therapy SSA SSA + Pegvisomant	11 4	26 4	21 3	OR = 0.2273 95% CI 0.03557–1.4522 *p* = 0.1174
Type of therapy Monotherapy Combination therapy	13 5	27 4	22 7	OR = 0.6261 95% CI 0.2936–2.766 *p* = 0.8557
Number of concomitant diseases ≤10 >10	18 5	34 3	30 5	OR = 0.72 95% CI 0.1918–2.7031 *p* = 0.6265
Age ≥65 years <65 years	5 19	14 24	9 24	OR = 0.7018 95% CI 0.2015–2.4444 *p* = 0.5780
TSA † Yes No	19 2	29 9	32 1	OR = 0.2969 95% CI 0.0252–3.4982 *p* = 0.3346

* statistically significant test result *p* < 0.05; ** due to lack of statistical and clinical significance for the results related to medium level of adherence, only the results for low and high level are presented; † TSA—Transsphenoidal adenectomy.

## Data Availability

The data that support the findings of this study are available from the corresponding author (M.K.), upon reasonable request.

## Supplementary Materials

The following supporting information can be downloaded at: https://www.mdpi.com/article/10.3390/pharmaceutics15020438/s1, The questionnaires (SF-36, EQ-5D and Morisky-Green 4-item) in Bulgarian and in English language.
