# Supplementary material for: Adherence to Acromegaly Treatment and Analysis of the Related Factors—A Real-World Study in Bulgaria
Source: Pharmaceutics. 2023 Jan 28;15(2):438. doi: 10.3390/pharmaceutics15020438 (PMC9963513; doi:10.3390/pharmaceutics15020438)
Supplement: Supplementary file 1 [file pharmaceutics-15-00438-s001.zip › pharmaceutics-2100383-supplementary.pdf]

In English language

1. Medication adherence

Morisky Medication-Taking Adherence Scale-MMAS (4-item)

(Please check one box on each line)

	Yes	*No*
1. Do you ever forget to take your (name of health condition) medicine?	o	o
2. Do you ever have problems remembering to take your (name of health condition) medication?	o	o
3. When you feel better, do you sometimes stop taking your (name of health condition) medicine?	o	o
4. Sometimes if you feel worse when you take your (name of health condition) medicine, do you stop taking it?	o	o

Source:

Beyhaghi H, Reeve BB, Rodgers JE, Stearns SC. Psychometric Properties of the Four-Item Morisky Green Levine Medication Adherence Scale among Atherosclerosis Risk in Communities (ARIC) Study Participants. Value Health. 2016 Dec;19(8):996-1001. doi: 10.1016/j.jval.2016.07.001. Epub 2016 Aug 31. PMID: 27987650; PMCID: PMC5287458.

2. Quality of Life Questionnaires

EQ-5D-3L

MOBILITY

I have no problems in walking about	☐
I have some problems in walking about	☐
I am confined to bed	☐

SELF-CARE

I have no problems with self-care	☐
I have some problems washing or dressing myself	☐
I am unable to wash or dress myself	☐

USUAL ACTIVITIES *(e.g. work, study, housework, family or leisure activities)*

I have no problems with performing my usual activities	☐
I have some problems with performing my usual activities	☐
I am unable to perform my usual activities	☐

PAIN / DISCOMFORT

I have no pain or discomfort	☐
I have moderate pain or discomfort	☐
I have extreme pain or discomfort	☐

ANXIETY / DEPRESSION

I am not anxious or depressed	☐
I am moderately anxious or depressed	☐
I am extremely anxious or depressed	☐

sample

Source: https://euroqol.org

- We would like to know how good or bad your health is TODAY.
- This scale is numbered from 0 to 100.
- 100 means the <u>best</u> health you can imagine.
  0 means the <u>worst</u> health you can imagine.
- Please mark an X on the scale to indicate how your health is TODAY.
- Now, write the number you marked on the scale in the box below.

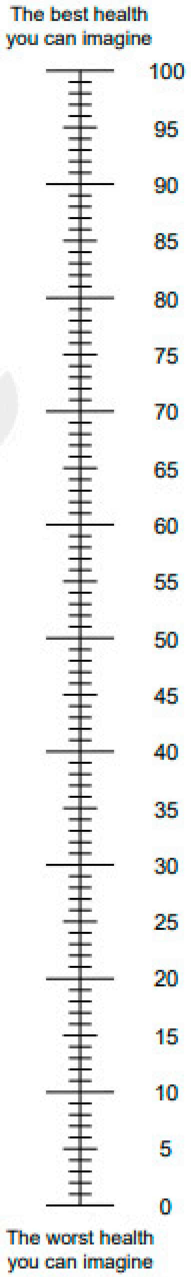

YOUR HEALTH TODAY = ☐

Sample

Source: https://euroqol.org

Short Form-36 (SF-36) Health Survey

1. In general would you say your health is:

• excellent [1]

• very good [2]

• good [3]

• fair [4]

• poor [5]

2. Compared to one year ago, how would you rate your health in general now?

• much better now than one year ago [1]

• somewhat better now than one year ago [2]

• about the same now as one year ago [3]

• somewhat worse now than one year ago [4]

• much worse now than one year ago [5]

3. The following items are about activities you might do during a typical day. Does your health now limit you in these activities? If so, how much?

a. Vigorous activities, such as running, lifting heavy objects, participating in strenuous sports.

b. Moderate activities, such as moving a table, pushing a vacuum cleaner, bowling, or playing golf.

c. Lifting or carrying groceries.

d. Climbing several flights of stairs.

e. Climbing one flight of stairs.

f. Bending, kneeling, or stooping.

g. Walking more than one mile.

h. Walking several blocks.

i. Bathing or dressing yourself.

Responses

• Yes, limited a lot [1]

• Yes, limited a little [2]

• No, not limited at all [3]

4. During the past 4 weeks, have you had any of the following problems with your work or other regular daily activities as a result of your physical health?

a. Cut down on the amount of time you spent on work or other activities.

b. Accomplished less than you would like.

c. Were limited in the kind of work or other activities.

d. Had difficulty performing the work or other activities (for example, it took extra effort).

Responses

• Yes [1]

• No [2]

5. During the past 4 weeks, have you had any of the following problems with your work or other regular activities as a result of any emotional problems (such as feeling depressed or anxious)?

a. Cut down on the amount of time you spent on work or other activities.

b. Accomplished less than you would like.

c. Didn't do work or other activities as carefully as usual.

Responses

• Yes [1]

• No [2]

6. During the past 4 weeks, to what extent has your physical health or emotional problems interfered with your normal social activities with family, friends, neighbors, or groups?

• not at all [1]

• slightly [2]

• moderately [3]

• quite a bit [4]

• extremely [5]

7. How much bodily pain have you had during the past 4 weeks?

• none [1]

• very mild [2]

• mild [3]

• moderate [4]

• severe [5]

• very severe [6]

8. During the past 4 weeks, how much did pain interfere with your normal work (including both work outside the home and housework)?

• not at all [1]

• a little bit [2]

• moderately [3]

• quite a bit [4]

• extremely [5]

9. These questions are about how you feel and how things have been with you during the past 4 weeks. For each question, please give the one answer that comes closest to the way you have been feeling. How much of the time during the past 4 weeks:

a. Did you feel full of pep?

b. Have you been a very nervous person?

c. Have you felt so down in the dumps that nothing could cheer you up?

d. Have you felt calm and peaceful?

e. Did you have a lot of energy?

f. Have you felt downhearted and blue?

g. Did you feel worn out?

h. Have you been a happy person?

i. Did you feel tired?

Responses:

• all of the time [1]

• most of the time [2]

• a good bit of the time [3]

• some of the time [4]

• a little of the time [5]

• none of the time [6]

10. During the past 4 weeks, how much of the time has your physical health or emotional problems interfered with your social activities (like visiting with friends, relatives, etc.)?

• all of the time [1]

• most of the time [2]

• some of the time [3]

• a little of the time [4]

• none of the time [5]

11. How TRUE or FALSE is each of the following statements for you?

a. I seem to get sick a little easier than other people.

b. I am as healthy as anybody I know.

c. I expect my health to get worse.

d. My health is excellent.

Responses

• definitely true [1]

• mostly true [2]

• don't know [3]

• mostly false [4]

• definitely false [5]

In Bulgarian language

1. ОЦЕНКА НИВОТО НА ПРИДЪРЖАНЕ КЪМ ТЕРАПИЯТА

ВЪПРОСИ:<br>(Моля, сложете отметка на верния отговор)	ОТГОВОРИ: ДА - 0 т.; НЕ - 1т.<br>0 - слабо придържане<br>1-2 - средно придържане<br>3-4 - високо придържане
Някога забравяли ли сте да приемате лекарството си?	ДА<br><br>НЕ
Нередовни ли сте в приема на лекарството си?	ДА<br><br>НЕ
Случвало ли Ви се е да спрете приема на лекарството си, когато се почувствате по-добре?	ДА<br><br>НЕ
Случвало ли Ви се е да спрете приема на лекарството си, когато се почувствате по-зле?	ДА<br><br>НЕ
РЕЗУЛТАТ:	

Адаптирано по Four-item Morisky Medication Adherence Scale (MGLS). Hadi Beyhaghi, MD, MPH. Psychometric Properties of the Four-item Morisky Green Levine Medication Adherence Scale among Atherosclerosis Risk in Communities Study (ARIC) Participants. Value Health. 2016;19(8):996-1001. doi:10.1016/j.jval.2016.07.001

2. <u>ОЦЕНКА КАЧЕСТВОТО НА ЖИВОТ НА ПАЦИЕНТА</u>

EQ-5D-3L

1. Двигателна активност
   A) Нямам проблеми при ходене
   B) Имам някои проблеми при ходене
   C) Прикован съм на легло

2. Самообслужване
   A) Нямам проблеми със самообслужването
   B) Имам някои проблеми при измиване и сресване
   C) Аз не съм в състояние да се мия и сресвам сам

3. Обичайни дейности (работа, учене, домашна работа, семейни или спортни дейности)
   A) Нямам проблеми в извършването на обичайни дейности
   B) Имам някои проблеми в извършването на обичайни дейности
   C) Не мога да извършвам обичайни дейности

4. Болка/дискомфорт
   A) Нямам болка или дискомфорт
   B) Имам поносими болки или дискомфорт
   C) Имам непоносими болки или дискомфорт

5. Подтиснатост/депресия
   A) Не съм подтиснат или депресиран
   B) Аз съм малко подтиснат или депресиран
   C) Аз съм много подтиснат или депресиран

Source: https://euroqol.org

За да помогнем на хората да кажат колко е добро или лошо е здравословното им състояние, ние направихме скала, на която най-доброто състояние, което можете да си представите е маркирано със 100 и най-лошото състояние, което можете да си представите, е маркирано с 0.

Бихме искали да посочите в този мащаб колко добро или лошо е вашето здраве днес по ваше мнение.
Моля, направете това, като начертаете линия върху скалата по-долу, в зависимост от това коя точка на скалата показва колко е добро или лошо здравословното ви състояние днес.

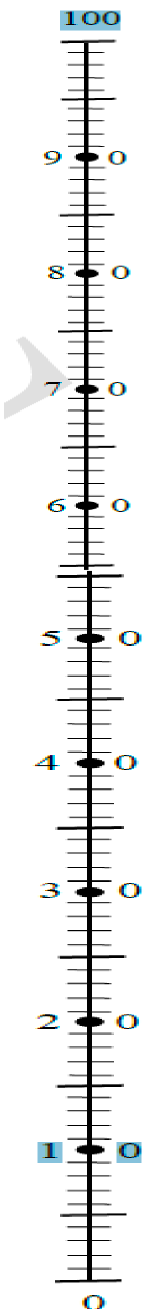

Source: https://euroqol.org

Анкета (SF-36) за оценка на качеството на живот

Изследването има за цел да разбере вашата гледна точка относно здравето ви. Тази информация ще помогне да се проследи как се чувствате и доколко сте способен да извършвате обичайните си дейности.

1. Като цяло бихте ли казали че здравето ви е:

- отлично [1]
- много добро [2]
- добро [3]
- средно [4]
- лошо [5]

2. В сравнение с предходната година, как бихте определили като цяло здравето си сега?

- Много по-добре сега отколкото преди 1 година [1]
- Малко по-добре, отколкото преди 1 година [2]
- Както преди 1 година [3]
- Малко по- зле отколкото преди 1 година [4]
- Много по- зле отколкото преди 1 година [5]

3. По-долу са изброени дейности, които бихте могли да извършвате през един обикновен ден.

Ограничава ли ви здравето в момента да извършвате тези действия и ако ДА до каква степен?

Дейности	Да, много ме ограничава	Да, малко ме ограничава	Не, не ме ограничава
Трудоемки дейности, като тичане, вдигане на тежки предмети,<br><br>участие в екстремни спортове.	1	2	3
Нормални дейности като преместване на маса, чистене с прахосмукачка, игра на тенис, футбол.	1	2	3
Носене или вдигане на тежести	1	2	3
Изкачване на няколко стъпала.	1	2	3
Изкачване на едно стъпало.	1	2	3
Навеждане или коленичене.	1	2	3
Ходене повече от един километър	1	2	3
Да извървите няколко пресечки.	1	2	3
Да се обличате или къпете.	1	2	3

4. През последните четири седмици имали ли сте някои от следните проблеми с изпълнение на работните си задължения или на обичайни дейности, като резултат от физическото ви състояние?

Дейности	Да	Не
Съкращавате време, което отделяте за работа или други занимания, поради физическо неразположение	1	2

Не ви удовлетворява постигнатото	1	2
Намалявате работа или други дейности, поради физическо неразположение	1	2
Имали сте затруднения при изпълнене на работа или други дейности (напр. коствало ви е повече усилия)	1	2

5. През последните четири седмици, имали ли сте някои от следните проблеми с работата си или с обичайните си дейности, като резултат от емоционални проблеми (депресия или напрегнатост)

Дейности	Да	Не
Съкратено време, което отделяте за работа или други занимания, поради психическо напрежение	1	2
Не ви удовлетворява постигнатото	1	2
Не сте работили или извършвали други занимания така внимателно както обикновено	1	2

6. През последните четири седмици до каква степен вашите здравословни и емоционални проблеми са повлиали на нормалните ви социални активности със семейството, приятелите, съседите или други групи?

а. Въобще не са ми повлияли [1]

б. Слабо [2]

в. До някъде [3]

г. Доста [4]

д. Изключително много [5]

7. Каква степен на физическа болка сте изпитвали през последните 4 седмици?

- Никаква [1] премини към въпрос 9
- Съвсем умерена [2]
- Умерена [3]
- Нормални [4]
- Силна [5]
- Много силна [6]

8. През последните четири седмици до каква степен болката е повлияла на нормалната ви работа (като работа извън къщи и домашна работа)

а. Въобще не ми е повлияла [1]

б. Слабо [2]

в. До някъде [3]

г. Доста [4]

д. Изключително много [5]

9. Тези въпроси оценяват как се чувствате сега и как сте се чувствали през последните 4 седмици. За всеки въпрос дайте отговор, който максимално се доближава до това как сте се чувствали и каква част от времето през последните 4 седмици:

Дейности	През цялото време	През значителна част от времето	През повечето време	От време на време	През малка част от времето	Изобщо
Чувствали сте се изпълнен със сила и енергия?	1	2	3	4	5	6
Били ли сте изнервен?	1	2	3	4	5	6
Чувствали ли сте се толкова депресира,че нищо не може да ви развесели?	1	2	3	4	5	6
Чувствали ли сте се спокоен и уравновесен?	1	2	3	4	5	6
Имали ли сте излишък от енергия?	1	2	3	4	5	6
Чувствате ли се подтиснат и тъжен?	1	2	3	4	5	6
Чувствали ли сте се изтощен?	1	2	3	4	5	6
Били ли сте щастлив?	1	2	3	4	5	6
Били ли сте изморен?	1	2	3	4	5	6

10. През последните четири седмици през каква част от времето вашето емоционално и физическо състояние е повлияло на вашите социални дейности (като посещение на приятели, роднини и т.н)

а. През цялото време [1]

б. През повечето време [2]

в. Понякога [3]

г. През малка част от времето [4]

д. Изобщо [5]

11. Верни или не са следните твърдения за вас?

Твърдение	Абсолютно вярно	До голяма степен вярно	Не знам	До голяма степен грешно	Абсолютно грешно
Изглежда се разболявам по-лесно от другите хора	1	2	3	4	5
Аз съм толкова здрав колкото и всеки останал, който познавам	1	2	3	4	5
Очаквам здравето ми да се влошава	1	2	3	4	5
Здравето ми е отлично	1	2	3	4	5
